# A Robust and Fast Method for Sidescan Sonar Image Segmentation Based on Region Growing

**DOI:** 10.3390/s21216960

**Published:** 2021-10-20

**Authors:** Xuyang Wang, Luyu Wang, Guolin Li, Xiang Xie

**Affiliations:** 1 School of Integrated Circuits, Tsinghua University, Beijing 100084, China; xuyang-w17@mails.tsinghua.edu.cn (X.W.); luyu-wan19@mails.tsinghua.edu.cn (L.W.); 2 Department of Electronic Engineering, Tsinghua University, Beijing 100084, China; guolinli@tsinghua.edu.cn

**Keywords:** segmentation, sonar images, fast and accurate, region growing

## Abstract

For high-resolution side scan sonar images, accurate and fast segmentation of sonar images is crucial for underwater target detection and recognition. However, due to the characteristics of low signal-to-noise ratio (*SNR*) and complex environmental noise of sonar, the existing methods with high accuracy and good robustness are mostly iterative methods with high complexity and poor real-time performance. For this purpose, a region growing based segmentation using the likelihood ratio testing method (RGLT) is proposed. This method obtains the seed points in the highlight and the shadow regions by likelihood ratio testing based on the statistical probability distribution and then grows them according to the similarity criterion. The growth avoids the processing of the seabed reverberation regions, which account for the largest proportion of sonar images, thus greatly reducing segmentation time and improving segmentation accuracy. In addition, a pre-processing filtering method called standard deviation filtering (*STDF*) is proposed to improve the *SNR* and remove the speckle noise. Experiments were conducted on three sonar databases, which showed that RGLT has significantly improved quantitative metrics such as accuracy, speed, and segmentation visual effects. The average accuracy and running times of the proposed segmentation method for 100 × 400 images are separately 95.90% and 0.44 s.

## 1. Introduction

Sidescan sonar (SSS), which can provide high-resolution images of the seabed, is one of the most common sensors for various underwater applications, such as topography measurement [1], search for sunken vessels and submerged settlements [2], underwater mine detection [3], fish stocks detection, cable or pipeline detection [4,5,6], and offshore oil prospecting [7]. Accurate and efficient segmentation of SSS images is essential for underwater objects detection. Because segmenting sonar images into highlight areas with objects, regions of shadow, and seafloor reverberation is an effective method to obtain the region of interest (ROI), this is usually an important step before object classification. However, the existing issues on sonar images such as low *SNR*, intensity inhomogeneity, and complex marine environment noise severely affect the performance of segmentation algorithms especially for small targets. In addition, the characteristics of sonar images are unstable, for example, shadows are not always present. In addition, shadows and highlight areas are affected by sonar position and the properties of the environment, which adds challenges for this task and makes it difficult to obtain a dataset that is robust for all environments to be encountered. Moreover, the algorithms should be rapid and efficient to enable real-time detection, but it is difficult to do this due to the high resolution of sonar images. This work concentrates on fast and accurate segmentation of sonar images into highlighted areas with objects, shadowed areas, and reverberant areas of the seafloor.

Various methods have been put forward to carry out sidescan sonar imagery segmentation. Widely used algorithms in the field of sonar image segmentation include threshold methods [8], clustering methods [9,10,11], Markov random field model (MRF) [12,13,14,15], curve evolution methods [16,17,18], convolutional neural networks (CNN) [19], etc. The threshold method usually faces the problem of varying illumination in sonar images, so it is difficult to find a suitable adaptive threshold. Methods based on clustering techniques (e.g., fuzzy C-means [11]) do not introduce modeling of the statistical distribution characteristics of luminance within the different types of regions, so these approaches lack robustness [15]. Active contour method [16,18] and level set method [17] based on curve evolution have been proposed and have been further investigated with good performance. MRF models are applied to sonar image segmentation and satisfactory results are achieved. However, these algorithms are quite complicated and require a large number of computational resources and running time [11], making them difficult to be applied for Autonomous Unmanned Vehicles (AUVs). Recently, CNN based methods have been researched on sonar segmentation [19]. However, the availability of a sufficient amount of training data to learn the classification model is still challenging, and the model results depend on the similarity between the training data and the test data. When the data are mismatched, the observed results may be inferior.

Filtering is commonly used to remove speckle noise to enhance the segmentation effect. Traditional image denoising algorithms can be generally divided into spatial domain and transform domain filtering methods. Lee [20], Frost [21], and SRAD [22] are typical spatial domain filtering methods with excellent filtering performance. However, the processed image appears too smooth and has blurred edges [23]. The transform domain filtering method contains common Discrete Cosine Transform [24], principal component analysis [25], and wavelet denoising algorithms [26]. Karthikeyan and Chandrasekar proposed a method combining the SRAD filter with the wavelet-based technique [27]. The method achieved a PSNR metric value of over 70 dB, as measured on a test dataset. Researchers used the stationary wavelet transform for sonar image denoising in [28], and the method has outstanding performance in terms of PSNR and SSIM. The transform domain filtering method can achieve a certain denoising effect, but it will remove the high frequency components of the signal itself at the same time, which results in detail loss [23].

In order to perform unsupervised segmentation effectively and efficiently, an algorithm based on region growing and a priori statistical distribution characteristics is proposed, called RGLT. The method first selects seed regions containing highlight and shadow areas and then grows them according to similarity criteria. Because the seabed reverberant areas take up the largest proportion of the sonar images while the highlight and shadow take up a small proportion, avoiding processing seabed areas when growing can save a considerable amount of time. The statistical characteristics are taken into account by a highlight-shadow likelihood ratio test at the stage of the seed regions selection, allowing the algorithm to efficiently and adaptively obtain preliminary segmentation. To solve the problem of low contrast, low *SNR*, and varying illumination, pre-process enhancement methods are presented to improve the contrast and *SNR* of a side scan sonar image, remove the speckle noise, and balance the illumination throughout the image. The results show significant gains in terms of quantitative metrics (speed and accuracy) and visual effects using RGLT. Compared with Fuzzy C-means [11] and active contour method [18], RGLT has obvious advantages in processing images with large differences in the ratio of background, highlight areas and shadows, and has better adaptability to noisy backgrounds and can clearly segment small targets.

The existing general issues on sonar images include low *SNR*, intensity inhomogeneity, and complex marine environment noise, which severely affect the performance of segmentation algorithms especially for small targets. In addition, most of the current methods with high accuracy and good robustness are iterative methods with high complexity and poor real-time performance.

The contributions of this article are summarized as follows:

1. This paper describes a pre-processing method for correcting time-varying gain (TVG) effects in sidescan sonar data, to balance the intensity throughout range.
2. To remove the speckle noise and enhance the *SNR* as well as the contrast of the sonar image, a filter *STDF* is proposed in this paper. The enhancement is beneficial to the subsequent segmentation.
3. An unsupervised segmentation algorithm is presented based on the assumption that the region of the seafloor obeys the Weibull distribution. Experiments showed the method has significantly improved quantitative metrics such as accuracy, speed, and segmentation visual effects. It is an effective and efficient algorithm based on region growing that can be used in the real-time sonar application.

## 2. Materials and Methods

### 2.1. Overview

**Figure 1:**
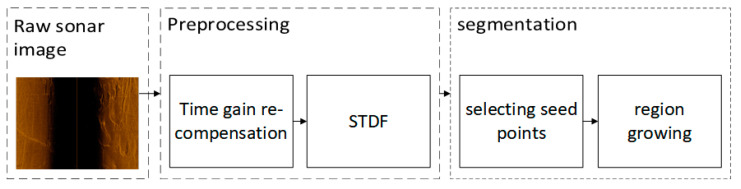
Block diagram of the proposed method process.

As illustrated in Figure 1, in this paper, the side-scan sonar images are processed in four steps. Firstly, the time gain of sonars is re-compensated in order to make the echo level independent of range. The step can alleviate the problem of varying illumination in sonar images and make the seabed intensity distribution in different regions consistent. It contributes to improving the robustness of adaptively seeds selection and region growing during subsequent segmentation. Sonar images suffer from low *SNR* and speckle noise, which adds difficulties to fine segmentation. Therefore, a filter referring as *STDF* is used to enhance sonar images and remove the speckle noise. Secondly, the ratio of the standard deviation to the mean (RDM) is a constant under the assumption that the sonar images obey the Rayleigh distribution. Exploiting the feature that the highlight regions have higher RDM than the seabed reverberation regions, *STDF* improves the *SNR* while removing speckle noise. To speed up the segmentation process for high-resolution sonar images, an algorithm based on regional consistency is proposed instead of the global iteration. To avoid losing the highlight and the shadow regions and to reduce the probability of the seabed regions being misclassified into the foreground, seed points are selected based on the statistical distribution characteristics and likelihood ratio test. The seed points are selected in the highlight and the shadow areas adaptively and efficiently to obtain the preliminary segmentation. Then, the subsequent growing is based on the preliminary segmentation and is conducted on the proposed similarity criterion. The seed selection enables the growth process to save lots of time without processing the seafloor reverberation region with large areas. The segmentation obtains fine details and high accuracy by the growth step.

### 2.2. Time Gain Re-Compensation

Time gain compensation (TGC) is indispensable in sonars in order to compensate for transmission loss and inconsistency of reverberation so as to make the echo level independent of range. The usual TGC will be embedded in the hardware according to the formula and then fine-tuned by manual adjustment. However, because the estimation of the parameters in the TGC formula is coarse (the parameters are related to the bottom substrate, geometric spreading type, water temperature, sonar frequency, etc.), the dependence of the intensity on the range cannot be completely eliminated, and the uniformity of the intensity is essential for the subsequent segmentation task, so a time gain re-compensation technique is proposed to reduce the dependence further.

When we consider the submarine interface reverberation, the received echo level, *E*, for an active sound system can be expressed as [29]:

(1) $$E=SL-\left(2TL+S_{s,v}+10\log\frac{c\tau }{2}\phi R\right)$$

where SL is the source level, TL is the one-way transmission loss, $S_{s,v}$ is the surface/volume backscattering intensity. c, τ, ϕ and R are speed of sound, width of transmitted pulse, horizontal opening angle of the transducer, and the range, respectively. The transmission loss is mainly caused by the geometric spreading and absorption of the acoustic. The commonly used function has the form 2TL(R)=40logR+2αR, where 40logR represents the transmission loss caused by the geometric spreading, and this function takes the assumption of spherical spreading in the free field. However, the sonar signal transmission does not always follow this ideal assumption. The function will be 20logR or 60logR, respectively, when taking the assumption of cylindrical spreading between parallel planes or hyperspherical spreading in free field with time expansion field. In addition, 2αR represents the transmission loss caused by the absorption of the acoustic, and α represents the logarithmic absorption coefficient. α is estimated on the experience usually, but the estimation is complicated and inaccurate, for that α is related to various factors such as sonar frequency, water temperature, salinity, and pressure.

From the above analysis, the gain compensation related to the range is abbreviated as follows:

(2) $$20\log\left(\frac{I\left(R_{ref}\right)}{I\left(R\right)}\right)=Gain\left(R,\lambda ,\alpha ,\gamma \right)=10\beta \log R+2\alpha R+\gamma$$

where $I\left(R_{ref}\right)$ and I(R) are the echo intensity at reference range $R_{ref}$ and range R. β and γ are constants. It is often the case that the image intensity still depends on the range due to imprecise parameters estimation in fact. Therefore, it is necessary to re-compensate the gain that has been compensated by hardware. In this paper, the parameters estimation problem is described as the nonlinear least squares problem as follows:

(3) $$\min\underset{R}{\sum }(20\log\frac{\tilde{I}\left(R_{ref}\right)}{\tilde{I}\left(R\right)}-Gain\left(R,\gamma ,\alpha ,C\right){)}^{2}$$

**Figure 2:**
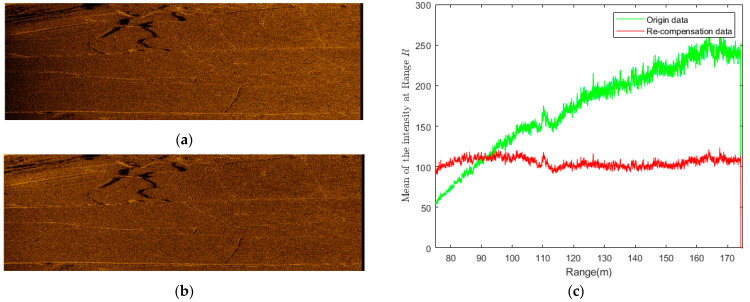
Comparison of a sonar image with and without time gain re-compensation. (**a**) A sonar image without time gain re-compensation. (**b**) A sonar image with time gain re-compensation. (**c**) The mean of the intensity at range R for origin data and re-compensated data.

Then, this problem is solved by the Gauss–Newton method to estimate parameters β, α, and γ. In the above equation, the mean of intensity at *R* and $R_{ref}$ ($i.e.,\;\tilde{I}\left(R\right)\;\mathrm{and}\;\tilde{I}\left(R_{ref}\right))$ obtained from data are used as the estimation of I(R) and $I\left(R_{ref}\right)$. The mean of the intensity at range R for origin data and re-compensated data are shown in Figure 2c. The re-compensation can balance the intensity inhomogeneity between the range. The intensity of sonar image without re-compensation is dependent on range obviously in Figure 2a. The sonar image with the presented re-compensation has a uniform intensity distribution over different ranges in contrast as shown in Figure 2b and can contribute to subsequent segmentation.

### 2.3. Filter for Enhancement

Since SSS systems generate images by coherent processing of the scattered signals, they are severely suffered from speckle noise [30]. Due to high levels of speckle noise, the underwater images often are generally poor quality with low *SNR* and contrast. Therefore, the pre-processing should be done to suppress noises and enhance the images before segmentation, feature extraction, and object detection. A pre-processing filter based on the statistical properties of the sonar scattering signal is proposed to remove the speckle noise. We called the proposed filter *STDF*.

#### 2.3.1. Speckle Noise Analysis

Speckle is a granular interference that inherently exists in and degrades the quality of the active radar, medical ultrasound, and sonar images [31]. In a sidescan sonar imaging system, the echo signal can be regarded as the coherent accumulation of backscatter signals generated by a large number of scattering points.

(4) $$Z=ue^{j\phi }=\overset{N-1}{\underset{i=0}{\sum }}z_{i}e^{j\theta_{i}}$$

where $Z=ue^{j\phi }$ is the overall impulse response of the SSS imaging system, and $z_{i}$, $\theta_{i}$ as well as N are the signal intensity, phase of the *i*th scattering point, and the number of scattering points within a resolution cell, respectively. If $\theta_{i}$ is a uniform distributed random variable taking values between 0 and 2π, and $z_{i}$ meets independent and identical distribution, the probability density function of u can be written as follows by using the central limit theorem [19]. where a is a constant:

(5) $$p\left(u\right)=\frac{u}{a^{2}}exp\left(-\frac{u^{2}}{2a^{2}}\right)$$

The ratio of the deviation σ and the mean μ (RDM) of the above distribution can be easily calculated as

(6) $$\frac{\sigma }{\mu }=\sqrt{\frac{2-\pi /2}{\pi /2}}\approx 0.523$$

Speckle is caused by the coherent accumulation of backscattered signals and can be modeled as multiplicative noises [20]. When the number of scatters which have random phases θ with uniform distribution tends to infinity, the above result will be obtained. The seabed reverberation areas are more consistent with the assumption than the highlight areas with targets. Experiments show that the RDM in the seabed reverberation areas is closer to the theoretical value of 0.523, and the RDM of the highlight areas is higher than that of seedbed reverberation areas. The RDM of highlight areas ($\mu_{h}/\sigma_{h}$) and seabed reverberation areas ($\mu_{b}/\sigma_{b}$) in 10 sonar images are calculated and shown in Table 1.

#### 2.3.2. The Proposed Filter *STDF*

The mean filtering can suppress the speckle noises and improve image quality by calculating the mean of the neighborhood to estimate the value of the centroid pixels [32]. As illustrated above, the mean of the neighborhood of a pixel can be estimated by estimating the standard deviation of the neighborhood, because the RDM is a constant. In our method, the deviation of each pixel is chosen instead of the mean of the pixel to enhance the targets and suppress speckle noise. The above description shows that the statistical properties of the highlight areas with targets are different from those of the seabed reverberation areas, i.e., the RDM of the highlight areas is higher than that of seabed reverberation areas. Therefore, using the standard deviation as an estimate for each pixel point can widen the margin between classes, amplify the signal of object areas, suppress the signal of seafloor background, and then improve the *SNR* and contrast of sonar images. The filter is called *STDF* and is defined as calculating the standard deviation of its 8-neighborhood as the estimated illumination of the pixel as $Y\left(x,y\right)=\sqrt{\frac{1}{n-1}\underset{s\in S}{\sum }{\left(J\left(x_{s},y_{s}\right)-\bar{J}\left(x,y\right)\right)}^{2}}$, where $J\left(x_{s},y_{s}\right)$ is the intensity at pixel $\left(x_{s},y_{s}\right)$ in the original sonar images. $\bar{J}\left(x,y\right)$ and n are the mean of pixels’ intensity and the number of pixels, in the 8-neigborhood S centering at (x,y). Y(x,y) is the *STDF* result at pixel (x,y). The filter can be calculated in matrix form and be expressed by Equation (7):

(7) $$I_{STDF}⨀I_{STDF}=\frac{1}{n-1}\left\{\left(I⨀I\right)⨂h\right\}+\frac{1}{n\left(n-1\right)}\left\{\left(I⨂h\right)⨀\left(I⨂h\right)\right\}$$

where I is the original sonar images, $I_{STDF}$ is the *STDF* result. h is a filter operator of size 3×3 whose elements are all 1. ⨂ indicates the convolution operation. ⨀ indicates the dot multiplication.

### 2.4. Proposed Segmentation

Because the sidescan sonar typically has a centimeter range resolution and a large range of hundreds of meters, there are numerous pixels in a single scan line. Most current segmentation (e.g., active contour [16,18], Fuzzy C-means [11], MRF [12,13,14,15]) are algorithms with high numbers of iterations and struggle to meet real-time requirements. Each iteration step of these algorithms involves the processing of reverberant regions of the seabed. The highlight regions with targets occupy a small proportion of the target, while the seabed reverberant regions occupy a large area. Aiming at concentrating on the highlight areas and shadow areas, the method based on seeded region growing (SRG) [33] is considered which focuses on the growth of the boundary pixels rather than on global processing. The region growing algorithm can correctly separate the regions that have similar properties as defined and is stable to noise. Yu et al. [34] proposes an image segmentation method that uses the region growing technique and edge strength information to improve the traditional MRF-based approaches. Jiao et al. [35] proposes an SRG algorithm with superior performance based on the Gaussian pyramid, which automatically selects seed points and optimizes the growth path. Wu et al. [36] proposes an effective segmentation method combined with MRF and region growing for sonar images. However, the speed of current region growing based methods combined with MRF does not meet the real-time requirement, and the region growing methods not employing the statistical features of sonar lack robustness.

The proposed segmentation method is composed of the following main steps: first, seed pixels or regions as started points are selected, similar to [33], and then, pixels with the same or similar properties in the vicinity of the seed pixel are merged into the seed pixel domain according to the growth criteria. In this paper, the seed selection principle and the similarity criterion for the region growing step are redesigned for segmenting sonar images fast and accuracy. The seeds are selected from the foreground (i.e., the highlight and shadow areas) to avoid the long-time iterative growth of the seabed reverberation area. The seed points are grouped into two sets $A_{i}$, i.e., the highlight areas $A_{1}$ and shadow areas $A_{2}$. The selection should be adaptive with no human interaction. Aiming at reducing the appearance of false targets without losing the foreground areas, every highlight region which needs to be extracted should have one seed point at least, and the probability that the seed points are in the seabed background area should be reduced as much as possible. Therefore, the method of likelihood ratio testing is adopted on the statistical distribution of the sonar images. Take the distinction between the seabed and the highlight points as an example. $y_{s}\in \left\{l_{0},l_{1}\right\}$ stands for the class of image points, and $l_{0}$ stands for the “highlight” label while $l_{1}$ corresponds to the “sea-bottom” class. Type I error is the rejection of a true null hypothesis, and type II error is failing to reject a false null hypothesis. The criterion for the selection of the rejection domain is to choose the rejection domain with the smallest possible probability of making the type II error while ensuring that the probability of making the type I error does not exceed a certain level. The criterion is consistent with the requirements of seed points selection.

According to the Section 2.3.1, the luminance within the seafloor reverberation area follows the Rayleigh distribution as Equation (5). In this section, we assume that the luminance within the seafloor reverberation area follows the Weibull distribution including the Rayleigh distribution, and the highlight and shadow areas follow the Gaussian law [15]. The conditional density function for the sea-bottom is modeled by a Weibull law and for highlight is modeled by a Gauss law as

(8) $$P(u|\lambda ,k,l_{1})=\{\begin{array}{c} \frac{k}{\lambda }{\left(\frac{u}{\lambda }\right)}^{k-1}e^{-{\left(\frac{u}{\lambda }\right)}^{k}}\;\;\;\;\;\;\;u\geq 0\\ 0\;\;\;\;\;\;\;\;\;\;\;\;\;\;\;\;\;\;\;\;\;\;\;\;\;\;\;\;\;\;\;\;u<0 \end{array}$$

(9) $$P(u|\sigma_{g},\mu_{g},l_{0})=\frac{1}{\sqrt{2\pi \sigma_{g}^{2}}}exp\left(-\frac{{\left(u-\mu_{g}\right)}^{2}}{2\sigma^{2}}\right)$$

where k, λ, $\sigma_{g}$, and $\mu_{g}$ are parameters to be estimated. To estimate the parameters for the seabed area luminance model precisely, pre-segmentation will be conducted using the threshold segmentation method with *STDF* images. The highlight regions, the shadow regions, and the seabed regions obtained by the pre-segmentation refer to the highlight data ℋ, the shadow data S, and the seabed data ℬ. The points numbers in three kinds of regions are $n_{ℋ}$, $n_{S}$, and $n_{B}$. The parameters of the Weibull model are estimated based on the raw data after time gain re-compensation but without *STDF*, as *STDF* will change the luminance distribution. The following expression of the maximum likelihood estimator can be obtained as follows, where $n_{ℱ}$ stands for $n_{ℋ}\;\mathrm{or}\;n_{S},$ and ℱ stands for ℋ or S.

(10) $$\hat{\lambda }={\left(\frac{1}{n_{B}}\overset{n_{B}}{\underset{i\epsilon ℬ}{\sum }}u_{i}{}^{k}\right)}^{\frac{1}{k}}$$

(11) $$\frac{1}{\hat{k}}=\frac{\sum_{i\epsilon ℬ}u_{i}{}^{k}\ln u_{i}}{\sum_{i\epsilon ℬ}u_{i}{}^{k}}-\frac{1}{n_{B}}\overset{n_{B}}{\underset{i=1}{\sum }}\ln u_{i}$$

(12) $$\hat{\mu_{g}}=\frac{1}{n_{ℱ}}\underset{i\epsilon ℱ}{\sum }u_{i}$$

(13) $${\hat{\sigma_{g}}}^{2}=\frac{1}{n_{ℱ}}\underset{i\epsilon ℱ}{\sum }{\left(u_{i}-\hat{\mu_{g}}\right)}^{2}$$

The likelihood ratio is calculated as

(14) $$\beta \left(u\right)=\frac{P(u|H_{0})}{P(u|H_{1})}=\frac{P(u|\sigma_{g},\mu_{g},l_{0})}{P(u|\lambda ,k,l_{1})}$$

The assumption test is as Equation (15), where η is the threshold for seed points selection. η is chosen by restricting the probability of making type I error $e_{1}$ to a certain percentage. Next, a test is chosen which minimizes class $e_{2}$. When β(u)≤η, pixels are assigned to the seed point sets; else, pixels are assigned to unallocated pixels.

(15) $$\beta \left(u\right)\begin{array}{c} \begin{array}{c} H_{0}\\ > \end{array}\\ \begin{array}{c} \leq \\ H_{1} \end{array} \end{array}\eta$$

The details of the process of region growing are shown in Appendix A. Firstly, the neighbors of the points in $A_{i}$ are marked as boundary pixels. Next, it is judged whether the bounding pixels set is empty. If the set is not empty, it is judged whether the points in the set satisfy the similarity criteria. The similarity criteria can be represented by the formula: $I_{n}\geq \bar{\mu_{B}}+\sigma_{B}$, where $I_{n}$ represents the intensity value of points in the boundary set. $\bar{I_{B}}$ and $\sigma_{B}$ represent the average intensity and the standard deviation of the intensity of the seabed reverberation area. If the points meet the similarity criteria, they are assigned to $A_{i}$, otherwise they are labeled as unallocated points. The iteration continues until the boundary set is empty, and all unallocated points are marked as seabed reverberation points.

## 3. Results and Discussion

The testing environment of the algorithm proposed in this paper is as follows: Intel (R) Core (TM) i58250U CPU, 8G RAM (Intel, Santa Clara, CA, USA), Windows, Matlab.

### 3.1. Dataset

In order to verify the effectiveness and superiority of the proposed algorithm, this paper conducts experiments on real sonar datasets. The seabed materials include sandy bottom and muddy bottom. The data are collected by a 500 kHz multi-beam sonar. Each scan line on each side covers a range of 175 m with 7000 pixels. The datasets contain complex landforms, such as terraces, ridges, ravines, gravel areas, etc.

### 3.2. Results of Proposed STDF

**Figure 3:**
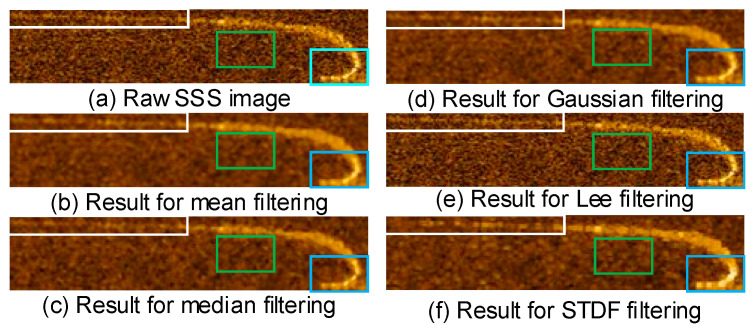
Results of the different filters. The subgraph (**a**) shows a raw SSS image without filtering, and subgraph (**b**–**f**), respectively, show the results for mean filtering, median filtering, Gaussian filtering, Lee filtering, and the *STDF* proposed in this paper.

**Figure 4:**
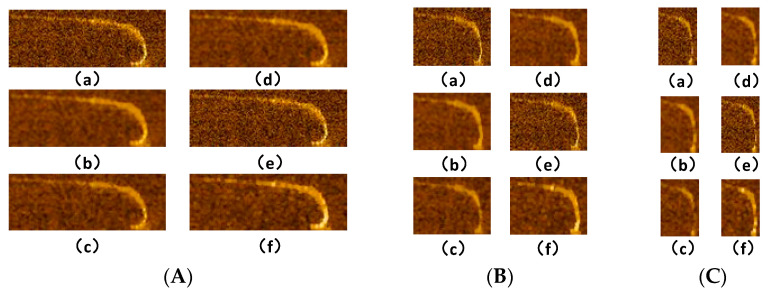
Results of different filters for downsampled sonar images. The column downsampling factors for subgraph (**A**), subgraph (**B**), and subgraph (**C**) are 2, 4, and 8, respectively. (**a**) Raw SSS image. (**b**) Results for mean filtering. (**c**) Results for median filtering. (**d**) Results for Gaussian filtering (**e**) Results for Lee filtering. (**f**) Results for proposed *STDF*.

Aiming at evaluating the performance of the *STDF*, the results are compared with mean filter, median filter, Gaussian filter, and Lee filter [20] in Table 2 in terms of *SNR* and contrast, defined as

(16) $$SNR=\left(I_{M}-\bar{I_{B}}\right)/\sigma_{B}$$

(17) $$contrast=\left(\bar{I_{H}}-\bar{I_{B}}\right)/\bar{I_{B}}$$

where $I_{M}$ is the maximum intensity value of the highlight areas; $\bar{I_{B}}$ and $\bar{I_{H}}$ represent the average intensity of the highlight areas and the seabed background areas, and $\sigma_{B}$ represents the standard deviation of the intensity of the seabed background area. The results demonstrate that the proposed *STDF* has higher *SNR* and contrast than other methods. The RDM of the highlight areas is higher than that of the seabed reverberation areas, thus calculating the standard deviation of its 8-neighborhood as the estimated illumination of the pixel can significantly improve *SNR* and contrast. The visual results of different filters are shown in Figure 3. The subgraph (a) shows a raw SSS image without filtering, and subgraphs (b), (c), (d), and (e) respectively show the results for mean filtering, median filtering, Gaussian filtering, Lee filtering, and the *STDF* proposed in this paper. The results in (b), (c), (d) suffer from details missing, namely, the tail of the cable in white boxes is not clear. The results in (e) still have obvious intensity inhomogeneity in the seabed area indicated by the green boxes. In addition, as illustrated in blue boxes, the results of *STDF* shown in (f) have better contrast of highlight areas with seabed areas than other methods, i.e., the *STDF* can improve the *SNR* and contrast of the sonar images meanwhile preserving details and edges. To verify the sensitivity characteristics with respect to the resolution of the sonar images, sonar images are downsampled in the column with the sampling rate reduction factors of 2, 4, and 8 in the column’s direction as shown in subgraphs (A), (B), and (C) in Figure 4, respectively. The subgraphs (a)–(f) in Figure 4 correspond to the same methods as Figure 3. As the downsampling rate increases, the resolution of the image decreases. It can be seen that the proposed *STDF* still has finer edges and higher contrast than other methods under low resolution conditions. It proves that the proposed method has low sensitivity.

### 3.3. Segmentation Results of Proposed Method

The method is compared with two robust unsupervised segmentation methods, namely, the fuzzy C-means clustering [11] and the active contour method [18]. The corresponding ground-truth segmentation maps of the real sonar images are obtained by manual segmentation. The segmentation accuracy ρ is used for the overall evaluation of the whole segmentation results, which is calculated as follows.

(18) $$\rho =N_{c}/N_{t}\times 100\%$$

where $N_{c}$ is the number of correctly segmented pixels, and $N_{t}$ is the total number of pixels. The ratio of correct to incorrect is defined as $\zeta =N_{c}/(N_{t}-N_{c})$.

**Figure 5:**
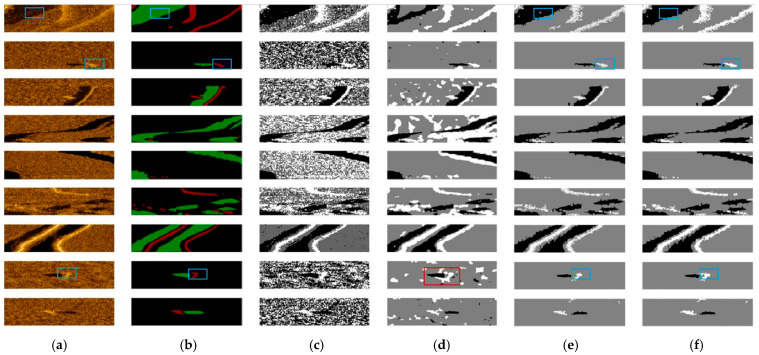
Segmentation results of the four methods: (**a**) The real sonar images. (**b**) The corresponding manually labeled ground truth segmented images. The green areas represent the shadow areas, and the red areas represent the highlighted areas. (**c**) The segmentation results of fuzzy C-means. (**d**) The segmentation results of the active contour. (**e**) The segmentation results of RGLT I. (**f**) The segmentation results of RGLT II.

The quantitative measures of the results are given in Table 3. Our proposed algorithm is named RGLT I (*w*/*o STDF*) or RGLT II (*w*/ *STDF*). Table 3 shows that the performance of both proposed methods RGLT I and RGLT II is better than that of the fuzzy C-means clustering and active Contour method in segmentation accuracy. In addition, the accuracy and the correct to incorrect ratio of RGLT II is higher than that of RGLT I, which indicates that *STDF* filtering not only contributes to the improvement of *SNR*, contrast, and visual effect but also improves the final segmentation effect significantly. Table 3 shows the average running times of the four segmentation methods used for 100 × 400 images segmentation. The proposed segmentation method is much faster than the fuzzy C-means and the active contour method. The data generation speed is 1 s/ping, and the speeds of RGLT I and RGLT II are 0.75 s/ping and 0.77 s/ping on average; thus, the proposed methods have an obvious advantage for real-time sonar detection. The results demonstrate that only growth in the highlight areas and shadow areas can effectively increase the speed of segmentation. Some segmentation results of the four methods are given in Figure 5 and are summarized in Table 4. As can be seen from Figure 5c, the fuzzy C-means method is sensitive to noise, and the problem of misclassifying the background into highlighted or shadowed areas occurs. As shown in Figure 5d, the active contour method appears to misclassify the background reverberant region into the highlight region when the area size of the three types of regions is very different, for example, when the bright region in the image is very small. In addition, for small targets, the segmentation results of the active contour method are not accurate enough, as shown in the red bounding box in Figure 5d. Figure 5e shows that our proposed RGLT I is robust to noise and has a low probability of background misclassification. This was a benefit from the low false alarm obtained by the maximum likelihood ratio test during the seed region selection phase. By comparing Figure 5e,f, it can be seen that the segmentation results are more accurate at the edges after using *STDF*. The details in the blue bounding box show that the growth of RGLT II is finer and more complete in the presence of noises than RGLT I. It shows that *STDF* can alleviate the interference of noise on the target edge.

## 4. Conclusions

In this paper, an unsupervised segmentation algorithm that using likelihood ratio testing is presented, which can rapidly and accurately segment noisy sonar images. Firstly, a time gain re-compensation method is used to balance the intensity throughout the range. Then a filter referring to as *STDF* is proposed in this paper to enhance the *SNR* and contrast of the sonar image. The results demonstrate that the proposed *STDF* has higher *SNR* (126.42) and contrast (1.36) than other methods. The enhancement can preserve details as well as edges and be beneficial to the subsequent segmentation. Lastly, the proposed segmentation method, RGLT, is applied with real sonar images. The results demonstrate that the proposed method achieved favorable performance in both visual quality and qualitative quality in comparison with other outstanding methods. The performance of RGLT is better than that of the fuzzy C-means clustering and active Contour method in segmentation accuracy (95.90) and the correct to incorrect ratio (23.39). As for the running time, the proposed segmentation method is much faster than other methods, and thus, the proposed method has an obvious advantage for real-time sonar detection. In the future, we will study the integration of RGLT and detection algorithms to achieve real-time and intelligent detection and identification on AUVs in search and rescue at sea. Future work also involves generalizing existing algorithms to other sonar images, such as forward-looking sonar and synthetic aperture sonar.

## Figures and Tables

**Table 1:** Ratios of mean and deviation of highlight areas and seafloor reverberation areas in 10 sonar images.

Sonar Images	$\sigma_{h}/\mu_{h}$	$\sigma_{b}/\mu_{b}$
Image 1	0.8926	0.5590
Image 2	0.9266	0.5622
Image 3	0.9957	0.5665
Image 4	0.8484	0.5582
Image 5	0.8347	0.5537
Image 6	0.8882	0.5648
Image 7	0.9822	0.5639
Image 8	0.9187	0.5762
Image 9	1.0921	0.5768
Image 10	0.9791	0.5766

**Table 2:** Comparison of the *SNR* and contrast of several filtering methods.

Method	*SNR*	Contrast
Raw image	110.97	1.09
mean filter	87.53	1.08
median Filter	83.84	0.99
Gaussian Filter	84.54	1.08
Lee Filter	113.17	1.07
*STDF*	126.42	1.36

**Table 3:** Comparison of the overall accuracy and speed using several segmentation methods.

Methods	Accuracy (%)	Correct to Incorrect Ratio	Running Time (s)
Fuzzy C-means	62.23	1.65	13.71
active contour	86.70	6.52	6.23
RGLT I	95.76	22.58	0.43
RGLT II	95.90	23.39	0.44

**Table 4:** Summary of the visual effects of the segmentation methods.

Methods	Visual Effects
Fuzzy C-means	Is sensitive to noise and has the problem of misclassifying the background into highlighted or shadowed areas.
active contour	1. Misclassify the background when the area size of the three types of regions is very different. 2. For small targets, the segmentation results of the active contour method are not accurate enough.
RGLT I	Is robust to noise and has a low probability of background misclassification.
RGLT II	The growth of RGLT II is finer and more complete in the presence of noises than RGLT I.

## Data Availability

No new data were created or analyzed in this study. Data sharing is not applicable to this article.

## Appendix A

**Algorithm A1** The pseudo-code for the proposed segmentation process.
**Seed selection based on likelihood ratio test**
**Input:** The original image and the pre-segmentation using the threshold method.
**Output:** foreground points set $A_{i}$ and sedbed reverberation points set.
**Begin:**
1: Estimate the distribution parameters using (10)–(13)
2: Calculate the likelihood ratio β(u) using (14)
3: **if** β(u)≤η **then**
4: Pixels are assigned to the seed points sets $A_{i}$;
5: **else**
6: Pixels are assigned to unallocated pixels.
7: **end if**
**Region growing**
8: Label boundary pixels set B and their candidate labels i(x)
9: **while** (B is not empty) **then**
10: **If** the points x in set B satisfy the similarity criterion **then**
11: Append x to $A_{i}$;
12: **else**
13: Append x to unallocated pixels set.
14: **end if**
15: Upate the Label boundary pixels set B and their candidate labels i(x)
16: **end while**
**End**
